# Spatial and space-time clustering of tuberculosis in Gurage Zone, Southern Ethiopia

**DOI:** 10.1371/journal.pone.0198353

**Published:** 2018-06-05

**Authors:** Sebsibe Tadesse, Fikre Enqueselassie, Seifu Hagos

**Affiliations:** 1 Institute of Public Health, College of Medicine and Health Sciences, University of Gondar, Gondar, Ethiopia; 2 School of Public Health, College of Health Sciences, Addis Ababa University, Addis Ababa, Ethiopia; Johns Hopkins Bloomberg School of Public Health, UNITED STATES

## Abstract

**Introduction:**

Spatial targeting is advocated as an effective method that contributes for achieving tuberculosis control in high-burden countries. However, there is a paucity of studies clarifying the spatial nature of the disease in these countries. This study aims to identify the location, size and risk of purely spatial and space-time clusters for high occurrence of tuberculosis in Gurage Zone, Southern Ethiopia during 2007 to 2016.

**Materials and methods:**

A total of 15,805 patient data that were retrieved from unit TB registers were included in the final analyses. The spatial and space-time cluster analyses were performed using the global Moran’s *I*, Getis-Ord $G_{i}^{*}$ and Kulldorff’s scan statistics.

**Results:**

Eleven purely spatial and three space-time clusters were detected (*P <*0.001).The clusters were concentrated in border areas of the Gurage Zone. There were considerable spatial variations in the risk of tuberculosis by year during the study period.

**Conclusions:**

This study showed that tuberculosis clusters were mainly concentrated at border areas of the Gurage Zone during the study period, suggesting that there has been sustained transmission of the disease within these locations. The findings may help intensify the implementation of tuberculosis control activities in these locations. Further study is warranted to explore the roles of various ecological factors on the observed spatial distribution of tuberculosis.

## Introduction

Ethiopia remains among high Tuberculosis (TB) endemic countries in the world, with an estimated annual incidence of 177 per 100,000 population [1]. TB places an extraordinary public health, financial and social burden in the country [2, 3]. It is one of the most important infectious diseases responsible as a leading cause of death and second cause of hospital admission [4, 5]. The patients face various levels of isolation and rejection, including loss of employment, reduced education opportunities, vulnerability to disability and divorce or spoiled marriage prospects [6]. Moreover, co-infection with Human Immunodeficiency Virus and the emergence of resistance to numerous anti-TB agents are recognized as increasing problems in the country [7, 1].

During the past two decades, Geographic Information Systems (GIS) and spatial statistics were used to detect spatial and space-time clustering of TB in many developing countries [8–12]. Several studies have been conducted at the national, regional and district levels to investigate the epidemiology of TB in Ethiopia [13–16]. Although these studies reveal helpful information for TB control programs, spatial context such as clustering patterns of the disease have rarely been taken into account. Spatial and space-time cluster analyses of TB may help public health officials discover the high-risk geographical areas and population groups that require targeted interventions [8–12]. Lack of such information may contribute for the partial effectiveness of TB control programs in Ethiopia, where the national TB control program implements a uniform approach to allocate resource across the regions.

This study aims to identify the location, size and risk of purely spatial and space-time clusters for high occurrence of TB in Gurage Zone, Southern Ethiopia during 2007 to 2016. The information may contribute to more effective budget allocation, active search for symptomatic patients, drug distribution, recruitment of skilled human resources, guiding the design of vaccination programs, community awareness creation through public health advocacy, and identifying factors behind the spread of the disease in high-risk areas.

## Methods and materials

### Study area

**Fig 1:**
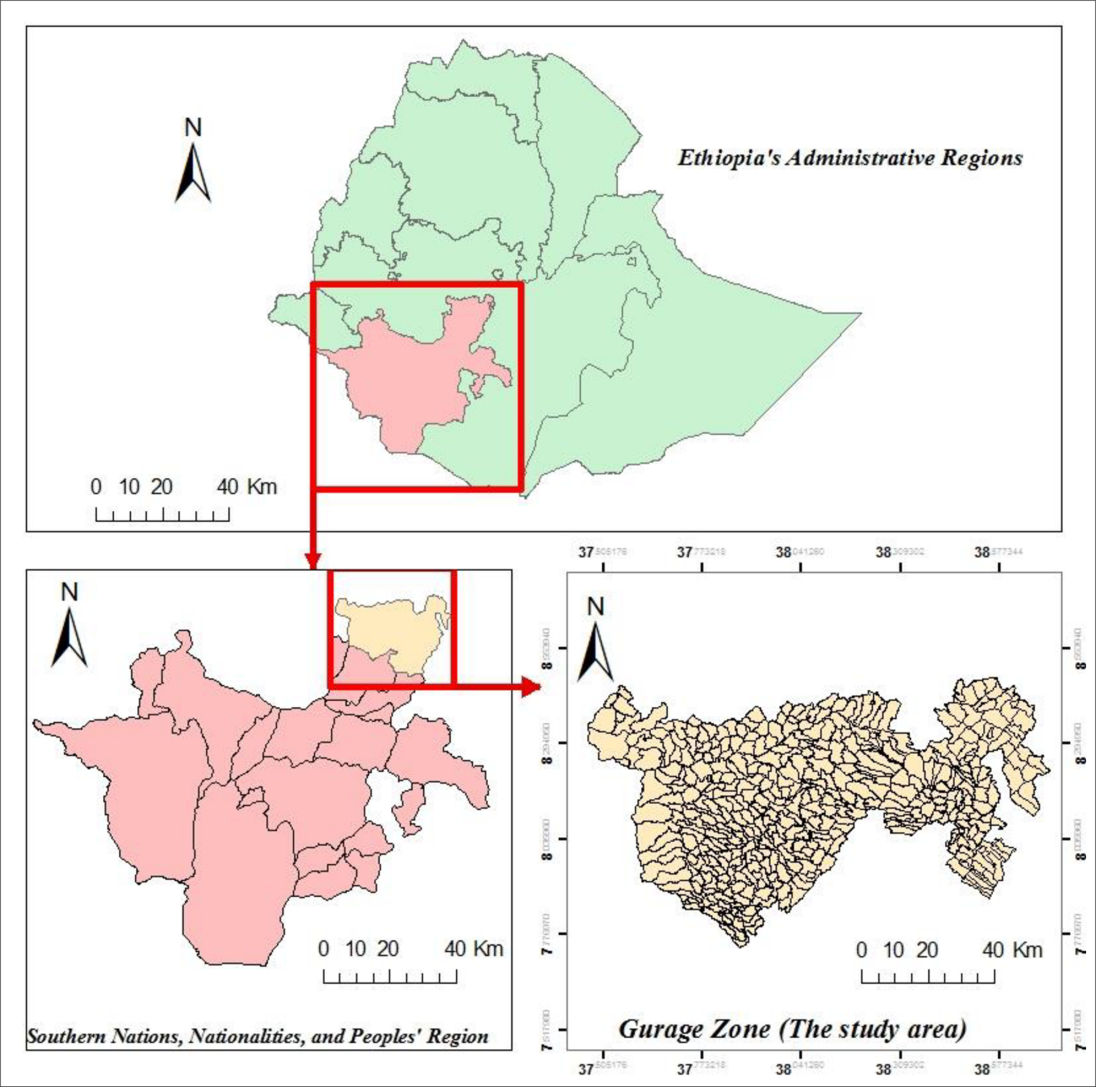
Map of the study area (Gurage Zone).

The study was conducted in the Gurage Zone, Southern Ethiopia, which is located between 7°76’ and 8°45’ N latitude and 37°46’ and 38°71’ E longitude **(Fig 1)**.The zone is divided into 13 districts and two town administrations (at Butajira and Wolkite).There are 403 rural and 20 urban kebeles (the smallest administrative units with a population of 5,000 on average) in the zone. About 84% of the populations live in the rural areas [17]. The zone has an average population of about 1,453,531 during 2007 to 2016 [18].

The zone has a total of 6 hospitals, 70 health centers, 414 health posts and 92 clinics that are involved in the prevention and control of TB. The health posts and clinics provide health education, identify suspects, refer patients to health centers and support treatment through trained health extension workers from the community. Health centers perform sputum microscopy, treatment and the referral of smear-negative and extra-pulmonary cases to hospitals for further management, while hospitals render diagnosis, treatment and inpatient care services [19].

The zone is selected for conducting this study mainly for two reasons. First, the zone has a geographic diversity characterized by mountain ranges and valleys. This may impact a geographic access to TB diagnosis and treatment centers [20–21]. Hence, it is assumed that TB cases are clustered in specific-geographic locations, and implementing a uniform TB control measures may not be effective in mitigating the problem. Second, according to the Southern Nations, Nationalities, and Peoples’ Regional State Health Department Report of 2016 the zone is among the highest hit areas by TB in the region [19].

### Data sources

The study was conducted from June to September, 2017. The list of DOTS-providing health facilities were obtained from the Gurage Zone Health Department database. Trained data collectors retrieved the patient information on sex, age, address, TB type, patient category and date of treatment started from unit TB registers at DOTS-providing health facilities in the Gurage Zone during 2007 to 2016. The patients’ addresses with similar names but from different geo-locations were linked to their correct geo-locations to prevent duplication. TB cases were diagnosed using pathogen detection, X-ray, and pathologic diagnosis according to the diagnosis criteria recommended by the national TB diagnosis and treatment guideline of Ethiopia [22]. The population and geo-location data for each kebele in the Zone were obtained from the Central Statistical Agency of Ethiopia (CSA).

#### Data quality control

The training of data collectors and supervisors emphasized issues such as data extraction format, field methods, and record keeping. The data were double entered and checked page-by-page by year, district, kebele and health facilities against unit TB registers for consistency and completeness throughout the entire data collection.

#### Data management and processing

Data entering, validating, cleaning and coding were employed using MS Excel (MicroSoft, Redmond, WA, USA). In the study area TB data were reported by the Basic Management Units. The reports might include cases outside of the administrative catchment or miss cases from their catchment enrolled in neighboring health facilities. The reason for this could be that the patients could cross the administrative boundaries for seeking health services by reason of access, preference and quality of care. Data aggregation based on the correct address of patients could help understand the true spatial nature of TB burden in the study area. Therefore, the patient data were aggregated at kebele level for spatial analyses in this study. Kebele centroids were used to represent a geographically weighted central location as coordinates.

### Spatial autocorrelation analyses

The global Moran’s *I* statistic was run in ArcGIS 10.2to examine the presence of spatial clustering of TB in the whole study area. The value of Moran’s *I* is calculated based on the deviation from the mean of two neighboring values. The following equation is used to calculate the Moran’s *I* statistic [8]:

$$I=\frac{n}{S_{0}}\frac{\sum_{i=1}^{n}\sum_{j=1}^{n}\omega_{i,j}z_{i}z_{j}}{\sum_{i=1}^{n}z_{i}^{2}}$$

where z_i_ is the deviation of a prevalence of TB for kebele *i* from its mean $\left(X_{i}-\bar{X}\right)$, z_j_ is the deviation of a prevalence of TB for kebele *j* from its mean $\left(X_{j}-\bar{X}\right)$, ω_i,j_ is the spatial weight between kebele *i* and *j*, *n* is the total number of kebeles, and *S*_0_ is the aggregate of all the spatial weights: $S_{0}=\sum_{i=1}^{n}\sum_{j=1}^{n}\omega_{i,j}$. The z_I_-score for the statistic is computed as: $z_{I}=\frac{I-E[I]}{\sqrt{V[I]}}$, where *E*[*I*] = −1/(*n*−1) and *V*[*I*] = *E*[*I*^2^]−*E*[*I*]^2^. The spatial relationships among kebeles were conceptualized by calculating the spatial weights from the input file containing the prevalence rate of TB for each kebele (the number of TB cases divided by the population of a given year and multiplied by 100,000) and the geo-coordinates data. A first order queen polygon continuity weights matrix, which defines the neighbors as those with either a shared border or vertex, was used for spatial weights [10]. The spatial weighing matrix was constructed in Geographical data analysis tool (GeoDa) by using the kebele-level polygon shape file Statistical significance for high occurrence of TB was decided when the Z-score ≥1.96 and a P-value ≤ 0.05.

### Purely spatial and space-time cluster analyses

The Kulldroff’s scan statistic was performed in SaTScan 9.2 to identify the location, size and severity of purely spatial and space-time clusters using the number of TB cases, the population for each kebele, the year of TB diagnosis and the geo-coordinates data as input files (**S1** and **S2 Tables)** [23]. The discrete Poisson model was used with the assumption that the number of TB cases at each location was Poisson distributed with a known population at risk. Scan circles of various sizes were used to identify the purely spatial clusters for high occurrence of TB. The upper limit for the maximum cluster size was set to 50% of the population at risk, which allowed small and large clusters to be detected. To identify space-time clusters for high occurrence of TB, a cylindrical window with the circular geographic base representing to the space and height to time was used. The size of the window was limited to 50% of the expected number of TB cases, and the time was set to the time period from 2007 to 2016. In both cases, the likelihood ratio was computed to measure a Relative Risk (RR) of TB occurrence within the cluster when compared to the risk outside using Monte Carlo simulations. The maximum number of replications for Monte Carlo simulation was set to 99,999. The cluster with the maximum Log Likelihood Ratio (LLR) was defined as the most likely cluster. The P-value was created using the combination of approximation [23]. A standard of ‘no geographical overlap’ was selected to report secondary clusters. Statistical significance was reported when a P-value was ≤ 0.05.

The Getis-Ord $G_{i}^{*}$ statistic was also implemented in ArcGIS 10.2 to identify the locations of clusters for high occurrence of TB. The $G_{i}^{*}$ statistic performs the spatial analysis by looking at each kebele within the context of a neighboring kebele. The local sum for a kebele and its neighbors is proportionally compared to the sum of all kebeles. When the local sum is much different than the expected local sum and that difference is too large to be the result of random chance, a statistically significant Z-score result. The following equation is used to compute the $G_{i}^{*}$ statistic [8]:

$$G_{i}^{*}=\frac{\sum_{j=1}^{n}\omega_{i,j}x_{j}-\bar{X}\sum_{j=1}^{n}\omega_{i,j}}{S\sqrt{\frac{\left[n\sum_{j=1}^{n}\omega_{i,j}^{2}-{\left(\sum_{j=1}^{n}\omega_{i,j}\right)}^{2}\right]}{n-1}}}$$

where *x_j_* is the prevalence of TB for kebele *j*, *ω_i,j_* is the spatial weight between kebeles *i* and *j*, *n* is the total number of kebeles, $\bar{X}=\frac{\sum_{j=1}^{n}x_{j}}{n}$, and $S=\sqrt{\frac{\sum_{j=1}^{n}{x_{j}}^{2}}{n}-{\left(\bar{X}\right)}^{2}}$. Therefore, the $G_{i}^{*}$ statistic is a Z-score. The spatial relationships among kebeles were conceptualized by calculating the spatial weights from the input file containing the prevalence rate of TB for each kebele and the geo-coordinates data. A first order queen polygon continuity weights matrix, which defines the neighbors as those with either a shared border or vertex, was used for spatial weights [10]. Statistical significance for high occurrence of TB was decided when the $G_{i}^{*}\geq 1.96$ and a P-value ≤ 0.05.

### Ethical considerations

The study protocol was reviewed and approved by the Research and Ethical Committee (REC) of the School of Public Health, and the Institutional Review Board (IRB) of the College of Health Sciences, Addis Ababa University. Since the study used data from the retrospective review of unit TB registers during 2007 to 2016, both the REC and IRB waived the requirement for informed consent from the patients. For this reason, informed consent was not obtained from the patients. A letter of support was obtained from the Gurage Zone Department of Health to obtain information from all districts and health facilities. The anonymity of cases was kept by using pseudo identification. Medical records were stored in a secure place to help maintain the confidentiality of the clinical information of cases.

## Results

### Demographic characteristics of TB cases

**Fig 2:**
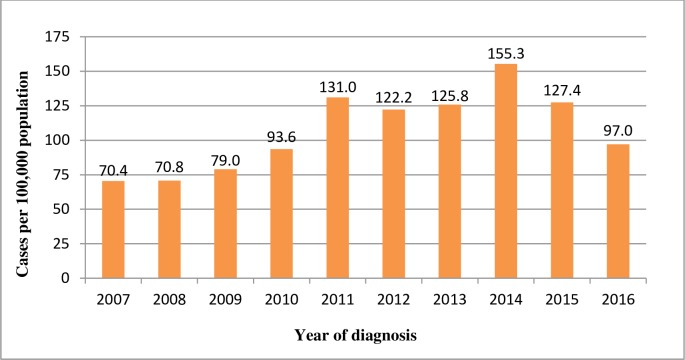
Trend of TB prevalence in Gurage Zone, Sothern Ethiopia, 2007–2016.

A total of 16,618 TB cases were diagnosed between 2007 and 2016. Of these, 4.9% were excluded from the final analyses due to incomplete addresses or being outside of the study area. Out of the 15,805 cases included in this study55.3% were males. The mean age with a standard deviation of the cases was 34.0±16.4. About 93.2% of the cases were newly diagnosed, while 6.8% were retreatment cases. Fifteen percent of the cases were from urban areas. The prevalence of TB varied from 70.4 to 155.3 cases per 100,000 population during 2007 to 2016 **(Fig 2)**.

### Spatial autocorrelation

The global Moran’s *I* statistic was significant for each year, implying that there were clusters in the distribution of TB in Gurage Zone, Southern Ethiopia **(Table 1)**.

**Table 1:** Global spatial autocorrelation of TB distribution in Gurage Zone, Southern Ethiopia, 2007–2016.

Year	Moran’s I	Z-score	P-value	Pattern
2007	0.266568	12.3	<0.001	Clustered
2008	0.143781	6.8	<0.001	Clustered
2009	0.194124	9.6	<0.001	Clustered
2010	0.288589	13.4	<0.001	Clustered
2011	0.191062	9.1	<0.001	Clustered
2012	0.161519	7.7	<0.001	Clustered
2013	0.116817	5.4	<0.001	Clustered
2014	0.130803	6.1	<0.001	Clustered
2015	0.174448	8.2	<0.001	Clustered
2016	0.098785	4.6	<0.001	Clustered

### Purely spatial clusters

**Fig 3:**
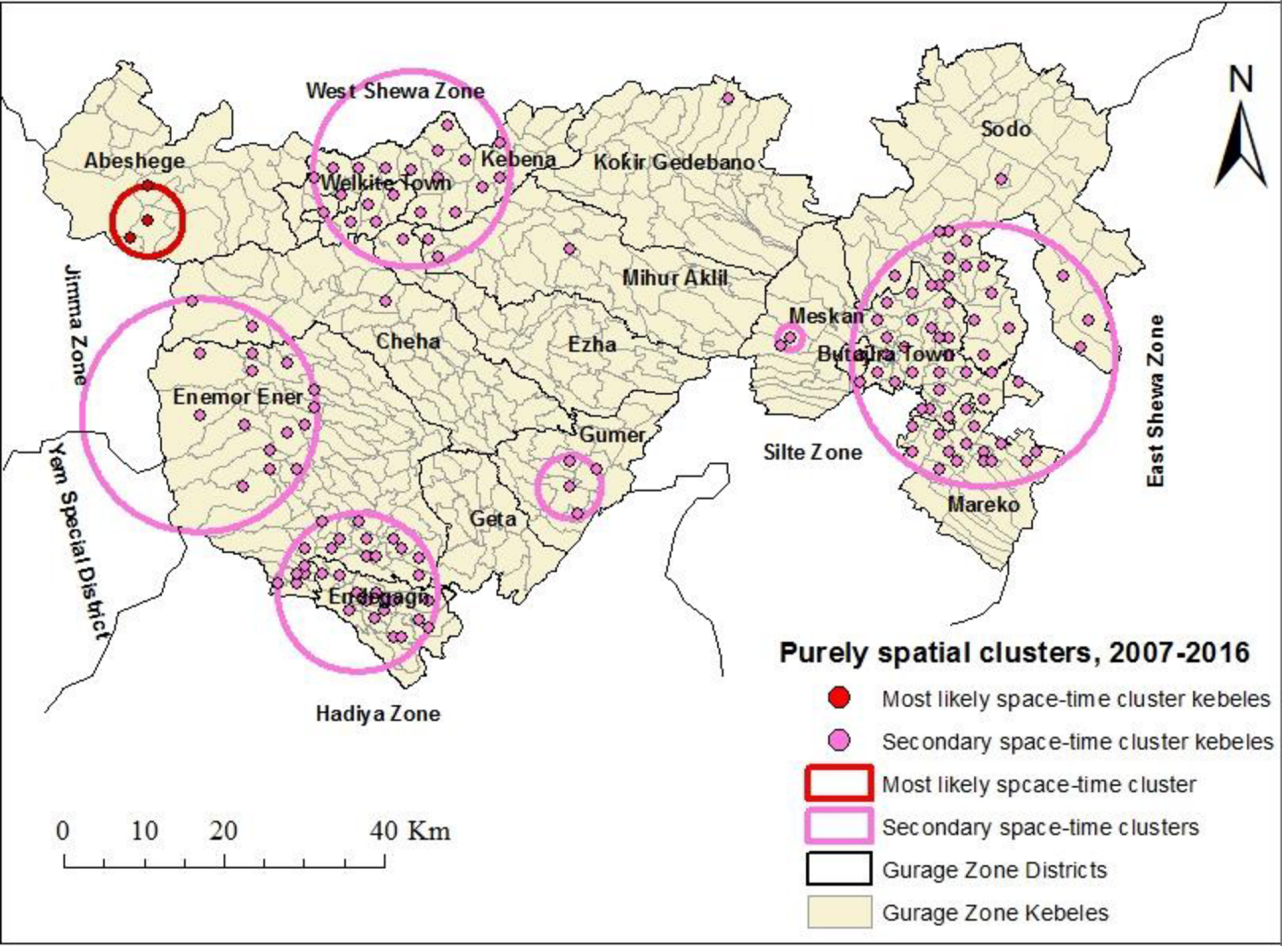
Significant purely spatial clusters for high occurrence of TB in Gurage Zone, Southern Ethiopia, 2007–2016.

The purely spatial cluster analyses identified the clusters for high occurrence of TB at peripheral areas of the geographic zone. The most likely cluster with 291 TB cases (71.19 expected cases) was detected at southwest of Abeshege district. The size of the cluster was within a radius of 4.45 km. People within this cluster had 4.16 times higher risk of TB infection than those outside the cluster. Eleven significant secondary clusters for high occurrence of TB were also detected. Most of these locations belong to the districts of Gumer, Endegagn, Enemor Ener, eastern Abeshege, Kebena, Welkite Town, northern Ezha, northwestern and eastern Cheha, Meskan, Butajira Town, northern Merako, southwest Sodo **(Table 2, Fig 3)**.

**Table 2:** Purely spatial clusters for high occurrence of TB in Gurage Zone, Southern Ethiopia, 2007–2016.

Cluster type	Cluster Year	Cluster center/radius	Observed cases	Expected cases	LLR	RR	P-value
Most likely cluster	2007–2016	(8.27 N, 37.58 E) / 4.45 km	291	71.19	192.87	4.16	<0.001
Secondary cluster	2007–2016	(8.12 N, 38.53 E) / 16.50 km	3325	535.65	135.75	1.39	<0.001
2^nd^ secondary	2007–2016	(7.85 N, 37.82 E) / 9.97 km	1365	998.14	64.99	1.40	<0.001
3^rd^ secondary	2007–2016	(8.33 N, 37.88 E) / 12.32 km	1253	942.62	49.53	1.36	<0.001
4^th^ secondary	2007–2016	(8.05 N, 37.64 E) / 14.69 km	685	460.81	49.21	1.51	<0.001
5^th^ secondary	2007–2016	(7.97 N, 38.06 E) / 3.98 km	340	213.02	32.51	1.61	<0.001
6^th^ secondary	2007–2016	(8.24 N, 38.06 E) / 0 km	36	8.93	23.14	4.04	<0.001
7^th^ secondary	2007–2016	(8.14 N, 38.31 E) / 1.56 km	218	136.27	20.91	1.61	<0.001
8^th^ secondary	2007–2016	(8.18 N, 37.85 E) / 0 km	58	23.55	17.86	2.47	<0.001
9^th^ secondary	2007–2016	(8.41N, 38.24 E) / 0 km	44	17.22	2.56	14.52	<0.001
10^th^ secondary	2007–2016	(8.32 N, 38.55 E) / 0 km	140	86.04	14.29	1.63	<0.001

**Fig 4:**
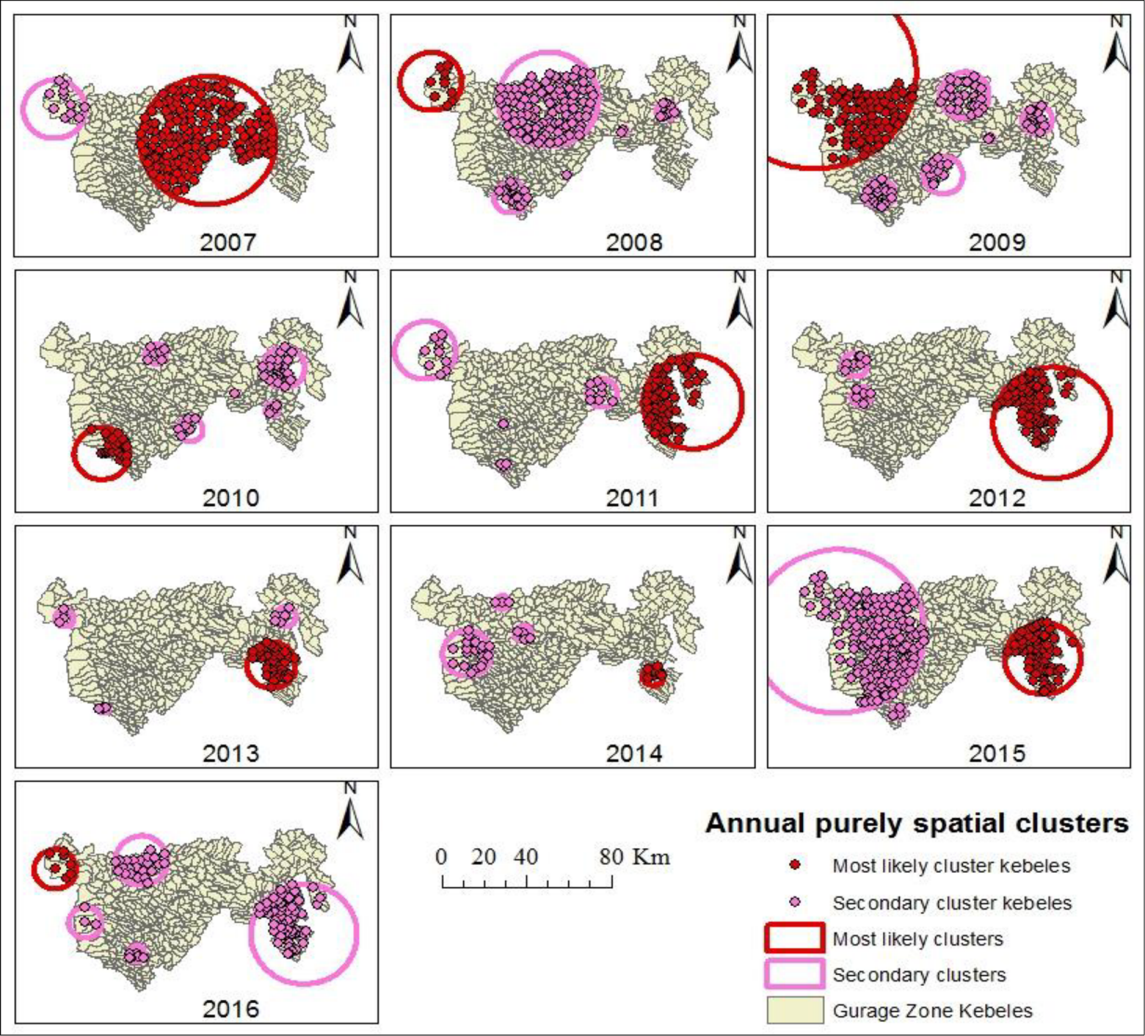
Annual purely spatial clusters for high occurrence of TB identified by using SaTScan statistic in Gurage Zone, Southern Ethiopia, 2007–2016.

**Fig 5:**
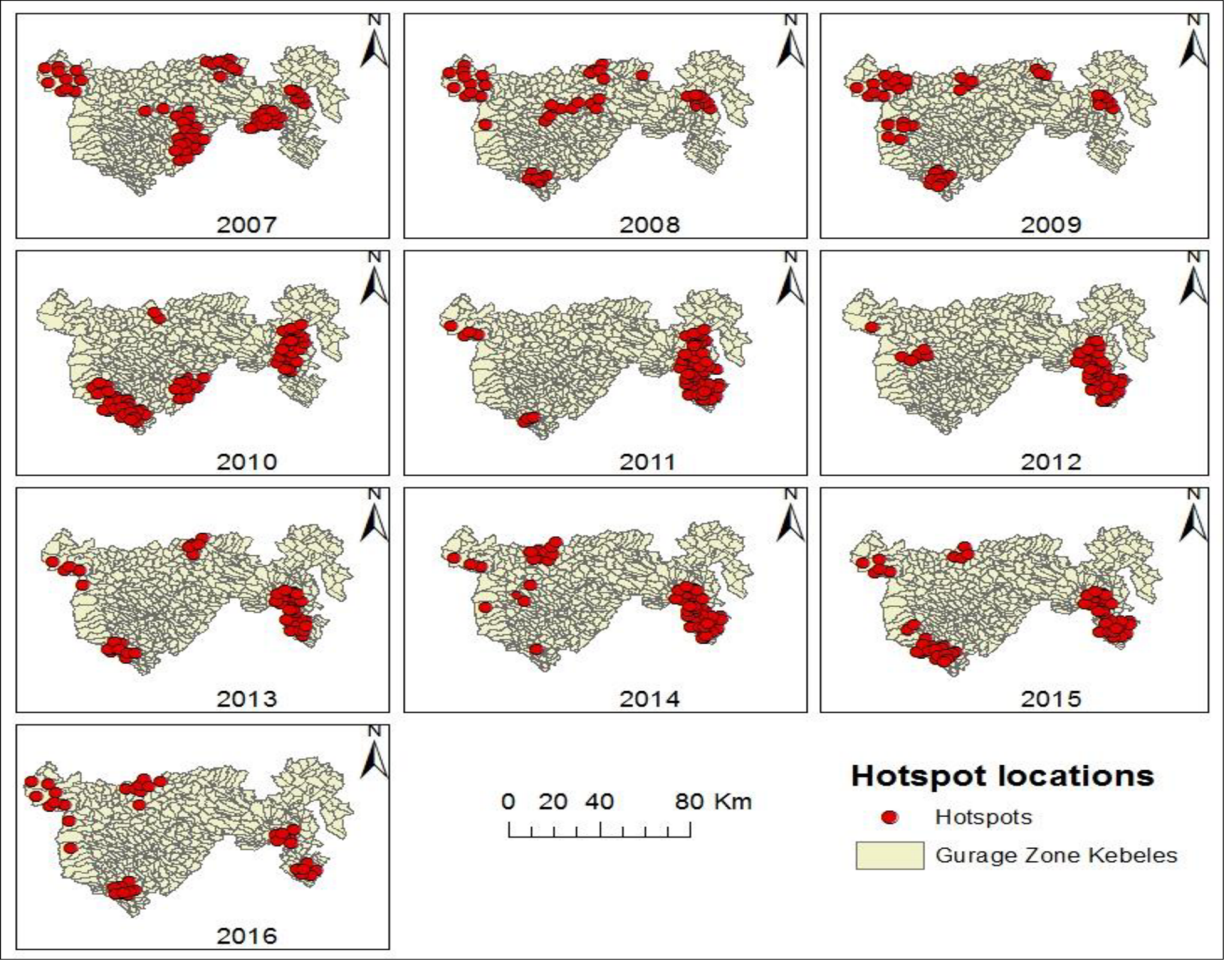
Spatial locations of significant hotspots of TB identified by using Getis-Ord $G_{i}^{*}$ statistic in Gurage Zone, Southern Ethiopia, 2007–2016.

The nature of the clusters for high occurrence of TB in the study area was evaluated for each year from 2007 to 2016. There were considerable spatial variations in the risk of TB by year **(Table 3, Fig 4).** Nearly consistent results were obtained by the $G_{i}^{*}$ statistic **(Fig 5)**.

**Table 3:** Annual purely spatial clusters for high occurrence of TB in Gurage Zone, Southern Ethiopia, 2007–2016.

Cluster type	Cluster Year	Cluster center/radius	Observed cases	Expected cases	LLR	RR	P-value
Most likely cluster	2007	(8.15 N, 38.17 E) / 32.07 km	635	406.44	118.53	2.91	<0.001
Secondary cluster	2007	(8.29 N, 37.51 E) / 14.61 km	86	24.92	47.64	3.71	<0.001
Most likely cluster	2008	(8.36 N, 37.49 E) / 14.45 km	101	22.78	75.66	4.85	<0.001
Secondary cluster	2008	(8.28 N, 37.99 E) / 23.45 km	325	215.73	32.70	1.78	<0.001
2^nd^ secondary	2008	(8.23 N, 38.49 E) / 4.54 km	44	12.91	23.40	3.53	<0.001
3^rd^ secondary	2008	(7.94 N, 38.07 E) / 0 km	14	2.13	14.56	6.65	<0.001
4^th^ secondary	2008	(7.84 N, 37.83 E) / 7.78 km	68	34.01	13.78	2.08	<0.001
5^th^ secondary	2008	(8.14 N, 38.31 E) / 1.56 km	25	8.04	11.55	3.17	<0.004
Most likely cluster	2009	(8.43 N, 37.56 E) / 47.88 km	382	194.53	92.08	2.50	<0.001
Secondary cluster	2009	(7.89 N, 37.84 E) / 6.97 km	85	39.00	21.26	2.28	<0.001
2^nd^ secondary	2009	(8.22 N, 38.51 E) / 7.02 km	61	26.13	17.44	2.42	<0.001
3^rd^ secondary	2009	(8.14 N, 38.31 E) / 1.56 km	28	9.20	12.53	3.10	<0.001
4^th^ secondary	2009	(8.33 N, 38.20 E) / 11.49 km	100	60.88	11.28	1.71	<0.005
5^th^ secondary	2009	(7.97 N, 38.11 E) / 9.16 km	71	39.17	10.90	1.87	<0.006
Most likely cluster	2010	(7.86 N, 37.73 E) / 12.99 km	137	54.72	46.24	2.68	<0.001
Secondary cluster	2010	(7.97 N, 38.11 E) / 5.98 km	89	31.16	36.90	2.99	<0.001
2^nd^ secondary	2010	(8.25 N, 38.51 E) / 10.06 km	111	52.84	25.61	2.20	<0.001
3^rd^ secondary	2010	(8.06 N, 38.46 E) / 3.13 km	60	21.73	23.24	2.85	<0.001
4^th^ secondary	2010	(8.13 N, 38.30 E) / 0 km	23	6.54	12.56	3.56	<0.001
5^th^ secondary	2010	(8.31 N, 37.96 E) / 4.93 km	35	14.97	9.85	2.38	<0.017
Most likely cluster	2011	(8.13 N, 38.64 E) / 23.49 km	507	267.67	103.35	2.23	<0.001
Secondary cluster	2011	(8.36 N, 37.49 E) / 14.45 km	109	45.68	32.59	2.47	<0.001
2^nd^ secondary	2011	(8.03 N, 37.83 E) / 0 km	26	7.19	14.71	3.65	<0.001
3^rd^ secondary	2011	(7.84 N, 37.83 E) / 1.56 km	27	7.84	14.32	3.48	<0.001
4^th^ secondary	2011	(8.17 N, 38.25 E) / 7.07 km	72	39.60	10.94	1.85	<0.006
Most likely cluster	2012	(8.00 N, 38.58 E) / 27.86 km	525	319.81	69.98	1.91	<0.001
Secondary cluster	2012	(8.12 N, 37.76 E) / 4.58 km	60	21.56	23.39	2.84	<0.001
2^nd^ secondary	2012	(8.26 N, 37.73 E) / 5.93 km	93	57.16	9.80	1.66	<0.018
Most likely cluster	2013	(8.06 N, 38.47 E) / 11.50 km	380	231.48	46.66	1.80	<0.001
Secondary cluster	2013	(7.86 N, 37.75 E) / 2.20 km	42	12.21	22.33	3.49	<0.001
2^nd^ secondary	2013	(8.28 N, 38.53 E) / 4.96 km	49	21.85	12.62	2.28	<0.001
3^rd^ secondary	2013	(8.27 N, 37.58 E) / 4.45 km	25	8.55	10.44	2.95	<0.001
Most likely cluster	2014	(8.00 N, 38.50 E) / 4.96 km	151	52.54	63.04	3.00	<0.001
Secondary cluster	2014	8.33 N, 37.85 E) / 3.30 km	48	11.27	33.11	4.33	<0.001
2^nd^ secondary	2014	(8.10 N, 37.70 E) / 11.50 km	144	87.60	15.87	1.68	<0.001
3^rd^ secondary	2014	(8.19 N, 37.94 E) / 3.98 km	46	22.48	9.53	2.07	<0.024
Most likely cluster	2015	(8.06 N, 38.51 E) / 17.92 km	481	334.77	34.58	1.57	<0.001
Secondary cluster	2015	(8.18 N, 37.63 E) / 40.94 km	843	688.91	25.27	1.38	<0.001
2^nd^ secondary	2015	(7.82 N, 37.89 E) / 3.13 km	43	18.53	11.88	2.35	<0.003
Most likely cluster	2016	(8.29 N, 37.51 E) / 9.92 km	66	23.11	26.96	2.94	<0.001
Secondary cluster	2016	(7.91 N, 37.86 E) / 3.98 km	57	21.01	21.32	2.78	<0.001
2^nd^ secondary	2016	(8.00 N, 38.58 E) / 25.06 km	343	251.21	18.24	1.47	<0.001
3^rd^ secondary	2016	(8.33 N, 37.88 E) / 12.32 km	148	95.13	13.49	1.61	<0.001
4^th^ secondary	2016	(8.05 N, 37.64 E) / 7.78 km	26	9.82	9.22	2.68	<0.030

### Space-time clusters

**Fig 6:**
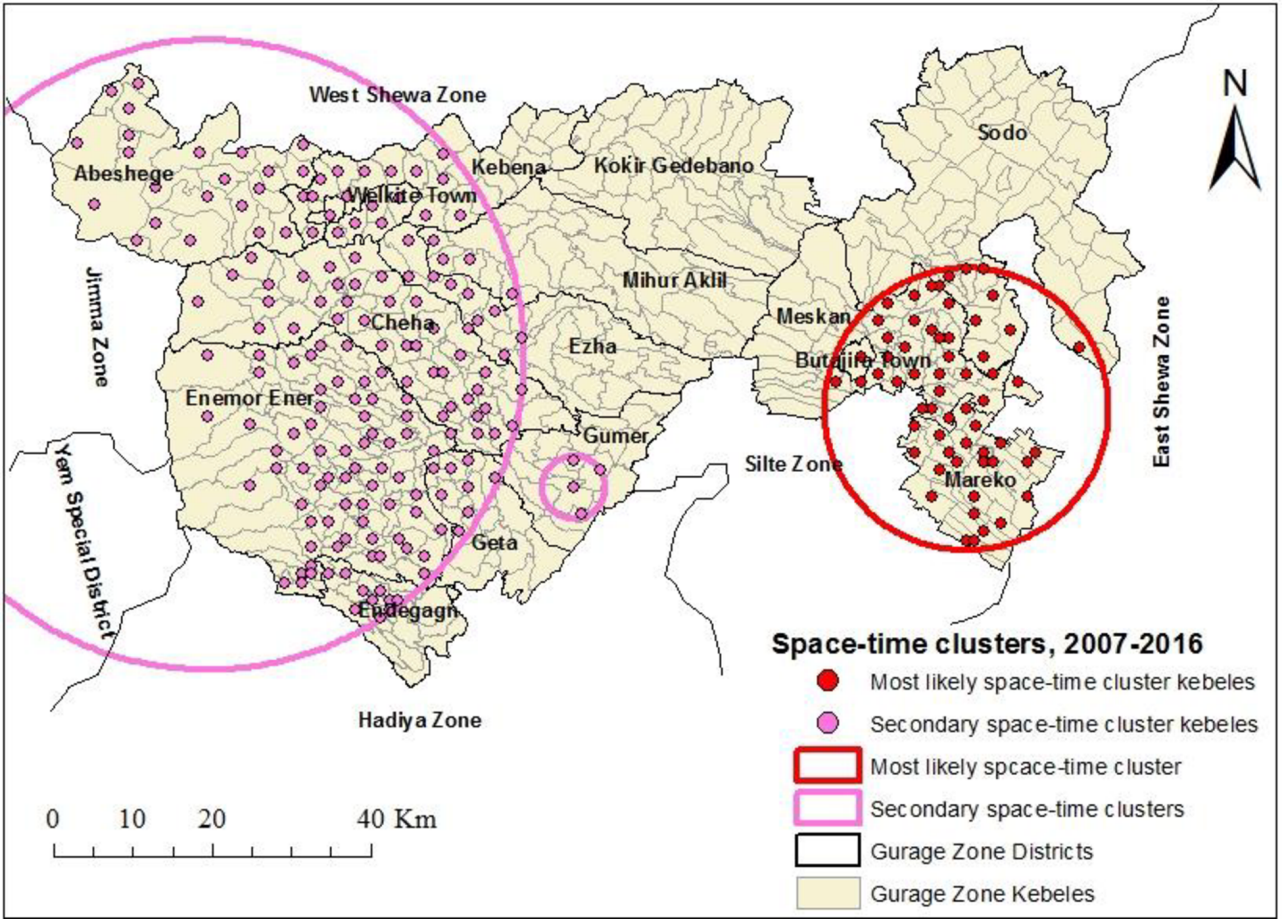
Significant space-time clusters for high occurrence of TB in Gurage Zone, Southern Ethiopia, 2007–2016.

The space-time cluster analysis identified the clusters for high occurrence of TB at peripheral areas of the study area. The most likely cluster with 2,502 observed cases (1,353.51 expected cases) was detected for the period 2011 to 2015.The cluster covered Meskan, Butajira Town, Merako and southwest of Sodo areas within a radius of 17.92 km. People living within this cluster had 2.01 times higher risk of TB infection than those outside the cluster. A secondary cluster covering Endegagn, Enemor Ener, Cheha, Abeshege, Welkite Town, weastern Kebena, northern Ezha and western Mihur Aklil areas was detected for the period 2011 to 2015. The second secondary cluster was detected at central Gumer district for the period 2007 to 2011 **(Table 4, Fig 6)**.

**Table 4:** Space-time clusters for high occurrence of TB in Gurage Zone, Southern Ethiopia, 2007–2016.

Cluster type	Cluster Year	Cluster center/radius	Observed cases	Expected cases	LLR	RR	P-value
Most likely cluster	2011–2015	(8.06 N, 38.51 E) / 17.92 km	2502	1353.51	435.61	2.01	<0.001
Secondary cluster	2011–2015	(8.12 N, 37.64 E) / 39.95 km	4117	3076.12	202.81	1.46	<0.001
2^nd^ secondary	2007–2011	(7.97 N, 38.06 E) / 3.98 km	197	99.21	37.64	2.00	<0.001

## Discussion

This study aims to identify the location, size and risk of purely spatial and space-time clusters for high occurrence of TB in Gurage Zone, Southern Ethiopia during 2007 to 2016. The clusters with high likelihood of TB occurrence were detected in border areas of the zone. The possible explanation for this could be that there were frequent cross-border population movements from the neighboring border areas of Jimma, Yem, Hadiya, Silte, West Shewa and East Shewa zones for economic and social reasons, which could favor the disease transmission in these areas. This is true according to other studies [24]. Therefore, future TB prevention and control efforts in these areas should include strengthening health infrastructure, staff capacity building, considering early diagnosis and treatment of symptomatic cases, and increasing community awareness. Furthermore, establishing a cross-border collaboration network may also help reduce the disease burden in these areas.

Three space-time clusters that were persistent for five years were detected in the zone. This might be due to the uniform implementation of TB prevention and control activities in the zone without targeting high-risk geographical areas [10]. Thus, application of GIS and spatial statistical techniques to identify purely spatial and space-time clusters for high occurrence of TB can be recommended for optimal utilization TB resources [25].

This study used the Kulldroff’s scan and Getis-Ord $G_{i}^{*}$ statistics to detect statistically significant clusters for high occurrence of TB in the Gurage Zone, Southern Ethiopia. The Kulldorff’s scan statistic is widely used in the field of public health to detect purely spatial and space-time clusters of infectious diseases, including TB [9–11]. The method searches for disease clusters without prior hypothesis on their location, size or time period. Moreover, Monte-Carlo randomization technique for hypothesis testing gives the empirical joint distribution of the statistics and hence accounts for the correlation among the statistics, providing a P-value after taking into account multiple testing [26]. In this and previous studies Kulldroff’s scan and Getis-Ord $G_{i}^{*}$ statistics have generated comparable results in identifying geographic areas where unusually high rates of TB occurred [10, 27]. However, some inconsistencies were observed which may be due to the fact that the methods used varying assumptions in determining clusters. The Kulldroff’s scan statistic calculated the maximum likelihood ratio of TB cases with relation to the underlying population in the area to detect larger clusters [23], whereas the Getis-Ord $G_{i}^{*}$ statistic examined each distance-defined grouping of values to identify more localized clusters [8]. Since there is no one gold standard method to detect disease clusters in spatial analyses it would be better to use more than one method at a time to cross-validate the results.

This study has some limitations. The study did not include TB patients who would remain undiagnosed, and those residents who were diagnosed and treated at health facilities outside the Gurage Zone. These could affect the nature of TB distribution by underestimating the prevalence. Another limitation of this study was that the Kulldroff’s scan statistic used circular spatial scanning windows and space-time cylinders with circular spatial bases which could not detect irregular shaped clusters, and could include a few non-significant locations. The annual projected population based on the 2007 census was used to provide up-to-date denominator population numbers which could be affected by uneven population growth across the kebeles. Besides, the confounding effects of covariates, like age and sex were not controlled in the spatial analyses since it was not possible to access the geo-coordinates data for the individual patients at the unit TB registers. This could also bias the results.

The strength of this study was that multiple methods were used to detect spatial clusters for high occurrence of TB in the study area. A high resolution spatial data were used to examine the spatial and space-time clustering of TB at the smallest administrative unit, which might be the best approach for TB control planning. A longer study period data were used to evaluate the changes in spatial and space-time clustering of TB. The study covered a wider geographical area containing urban and rural areas. Errors related to the geo-coding of cases were avoided by linking each case to the correct home address using geo-codes from the CSA. The spatial data included all forms of TB to explore the high-risk geographical locations which require more focused public health attention.

## Conclusions

This study showed that TB clusters were mainly concentrated at border areas of the Gurage Zone, suggesting that there has been sustained transmission of the disease within these locations. The findings may help intensify the implementation of TB control activities in these locations. Further study is warranted to explore the roles of various ecological factors on the observed spatial distribution of TB.

## Supporting information

S1 Table Data for spatial cluster analyses. (XLSX)

S2 Table Data for space-time cluster analyses. (XLSX)
